# The moderating role of hair cortisol in the association of early and recent stress with stress-related phenotypes

**DOI:** 10.3389/fpsyg.2023.1150142

**Published:** 2023-06-21

**Authors:** Pilar Torrecilla, Neus Barrantes-Vidal

**Affiliations:** ^1^Departament de Psicologia Clínica i de la Salut, Facultat de Psicologia, Edifici B, Universitat Autònoma de Barcelona, Barcelona, Spain; ^2^CIBER de Salud Mental, Instituto de Salud Carlos III, Madrid, Spain

**Keywords:** hair cortisol, psychosocial stress, life events, early adversity, schizotypy, anxiety, depression

## Abstract

**Background:**

Increased hair cortisol concentrations (HCC) have been found in clinical samples of schizophrenia, first episode psychosis and clinical risk for psychosis, but evidence of such is scarce in schizotypy. High HCC are supposed to reflect elevated chronic stress. However, HCC were not directly associated with adversity measures and stress-related phenotypes in previous research. This study tested whether HCC moderated the association between a comprehensive range of psychosocial stressors with several stress-related phenotypes in a sample of nonclinical young adults. It was expected that stressors, either distal (i.e., early-life) or recent, would be associated with subclinical features, particularly for those with elevated HCC, reflecting the effects of a potential biological sensitization to stress.

**Methods:**

The sample comprised 132 nonclinical young adults belonging to the Barcelona Longitudinal Investigation of Schizotypy Study (BLISS). Participants completed a questionnaire of childhood adversity and two complementary measures of recent life events, tapping threatening vs. more general life events. Both the frequency and subjective impact (positive vs. negative) of general life events were also assessed. Psychotic (i.e., schizotypy, suspiciousness) and non-psychotic (i.e., depression, anxiety) subclinical features as well as appraisals of perceived stress were examined. Hierarchical linear regressions and simple slope analyses were computed.

**Results:**

HCC moderated the effects of both early and recent stress on suspiciousness as well as the effects of recent life events on perceived stress, such that those with higher HCC presented increased suspiciousness and perceived stress at higher levels of stress exposure. Positive, but not negative, recent life events were associated with decreased perceived stress and depression, and these associations were moderated by low HCC, indicating a buffering effect for those with a non-impaired HPA axis.

**Conclusion:**

In line with the neural diathesis-stress model, results highlight the role of the interplay between the HPA axis and exposure to stressful experiences in exacerbating psychosis features and extend evidence to the nonclinical expression of the psychosis continuum. In addition, findings support the protective effect of positive experiences in decreasing stress appraisals and affective disturbances, which is consistent with emerging research about the relevance of positive factors in reducing the likelihood of psychopathological outcomes.

## 1. Introduction

The classic neural diathesis-stress model focuses on the role of the hypothalamic-pituitary-adrenal (HPA) axis as a mediator of the effects of stress on triggering and exacerbating psychosis symptoms (Walker and Diforio, 1997; Walker et al., 2008; Pruessner et al., 2017). The HPA axis acts in response to stressful situations or threats by activating a hormonal cascade that culminates in the release of the glucocorticoid cortisol to facilitate physiological and behavioral responses to threats. This response is suppressed by a negative feedback loop once stressors are absent. Cortisol is the most frequently used measure to study the HPA axis function and its implication in mental disorders as it can be easily assayed in blood, urine, saliva and hair. Whereas samples of blood serum and saliva have been extensively used to analyze acute single point cortisol responses to stress and urine to obtain short term levels (i.e., 24 h), hair cortisol concentrations (HCC) have been proposed as a more accurate measure of chronic stress enabling retrospective and long-term examination of cortisol production (Russell et al., 2012). As hair grows approximately 1 cm per month (Wennig, 2000), a 3 cm-hair sample for instance allows to retrospectively assess hair cortisol from the last 3 months from the strand collection providing a valid, reliable, non-invasive, and easily transported and stored long-term measurement of the stress glucocorticoid (Stalder et al., 2017). However, very few studies have examined HCC across the psychosis spectrum, reporting elevated levels in clinical samples of schizophrenia (Streit et al., 2016; Aas et al., 2019), First Episode of Psychosis (FEP) (Andrade et al., 2016) and populations at clinical risk for psychosis (Söder et al., 2019). To the best of our knowledge only one study (Torrecilla and Barrantes-Vidal, 2021) has examined HCC at the nonclinical end of the extended psychosis phenotype, that is, schizotypy, failing to find any significant association between HCC and a wide range of stress-related phenotypes. Schizotypy is conceptualized as a multidimensional construct indexing liability for psychosis-spectrum psychopathology that is expressed across a broad range of personality, subclinical and clinical psychotic features (Ettinger et al., 2014; Kwapil and Barrantes-Vidal, 2015; Grant et al., 2018a). Investigating HCC in nonclinical individuals with schizotypy traits allows examining the role of HPA axis in the etiology of psychosis without the confounds associated with clinical status and medication, which have commonly been related to inconsistent replication findings when examining baseline cortisol levels in psychosis populations (Pruessner et al., 2017).

It is well established that psychotic spectrum disorders are associated with childhood adversity (Mondelli and Dazzan, 2019; Sideli et al., 2020) and heightened stress-sensitivity (Myin-Germeys and van Os, 2007; Van Winkel et al., 2008; Cristóbal-Narváez et al., 2016; Rauschenberg et al., 2017). A stress sensitization mechanism seems to underlie this association; that is, prolonged and/or severe stressful experiences in highly sensitive developmental periods (i.e., childhood) might result in a process of behavioral and biological sensitization to stress through the disruption of stress regulation systems such as the HPA axis (Read et al., 2001; Yuii et al., 2007; Belda et al., 2015). This mechanism would enhance stress sensitivity by increasing physiological (e.g., cortisol secretion) and behavioral (e.g., greater intensity of stress appraisals) responsivity to subsequent minor adversities in adulthood, which has been associated to a broad range of phenotypes (McLaughlin et al., 2010; Hammen, 2015; Bandoli et al., 2017), including psychosis (Collip et al., 2008; Lataster et al., 2012). Although some evidence of an association between early adversity and altered HCC has been found in clinical psychosis (Aas et al., 2019; Schalinski et al., 2019) and clinical at-risk individuals (Söder et al., 2019), recent meta-analytic work by Cullen and colleagues (Cullen et al., 2020) found poor concordance between psychosocial stressors (including both early adversity and recent stressful events) and cortisol levels (including studies using all types of measurements) in psychosis and high-risk individuals. In previous work, we also failed to find a direct association between HCC and early adversity as well as frequency and subjective appraisals of recent life events in nonclinical young adults with a wide distribution of schizotypy (Torrecilla and Barrantes-Vidal, 2021).

The lack of overall group-level association between HCC, stress-related phenotypes, and exposure to early and recent stress in our previous study might be influenced by limited statistical power as well as the fact that nonclinical samples do not generally present very high or persistent levels of stress (Torrecilla and Barrantes-Vidal, 2021). However, drawing from the stress sensitization framework, we propose that this may also be because the association between both early and recent experiences of stress with subclinical psychopathology would be moderated by HCC. Support for the moderating role of HCC on the relationship between stressful experiences and stress-related phenotypes has been found, for instance, in nonclinical samples of children (Fuchs et al., 2018), adolescents (Shapero et al., 2019; Xu et al., 2019) and in clinical depression (Duncko et al., 2019), but little work has been conducted across the extended psychosis spectrum phenotype despite of considerable research linking psychosis vulnerability to HPA axis hyperactivation. Of note, there is a notable lack of research examining the psychobiological effects of exposure to stress transphenomically, even though recent claims from transdiagnostic approaches recognize the multidimensionality and evolving nature not only of a single diagnostic spectra (e.g., psychosis) but also across the whole psychopathology spectrum -including nonclinical manifestations- (van Os, 2015; McGorry et al., 2018), and ample evidence suggests that HPA axis disruption is present in many psychopathology phenotypes (Naughton et al., 2014; Herane-Vives et al., 2020; Koumantarou Malisiova et al., 2021).

The goal of the present study was to examine whether HCC moderate the association between a comprehensive range of psychosocial stressors, including both early and recent stressful experiences, with the expression of several stress-related phenotypes in a sample of nonclinical young adults. It was hypothesized that the association between early-life and recent stressors with subclinical features would be greater for those with elevated HCC. Specifically, we expected that the moderating role of the HPA axis biological indicator (i.e., HCC) would be relevant for all the phenotypes that have been robustly associated to stress, that is, the paranoid, positive schizotypy, depression and anxiety dimensions, as well as levels of perceived stress (e.g., Velikonja et al., 2015; Gibson et al., 2016; Bandoli et al., 2017; Anderson et al., 2022), but not with negative schizotypy. Furthermore, we expected that among recent life events the measure of threatening events would show a greater effect compared to that of general life events given their clear negative or even devastating nature. For the measure of recent general life events, we hypothesized that the subjectively rated impact of life events would yield more interaction effects than the raw number of events endorsed. Thus, subjective ratings of negative life events were supposed to increase the presence of subclinical phenotypes in those with higher levels of HCC, whereas those experiencing a greater positive impact from life events were expected to show a buffering effect by decreasing levels of subclinical features.

Several features of this study overcome limitations reported in previous work that have been suggested as possible sources of conflicting results. We investigated the effects of different time frames (early vs. recent), types (common vs. uncommon) and not only frequency but also subjective appraisals (positive vs. negative) of stressors in the same sample. Furthermore, unlike most previous studies, we tested the hypothesis across psychosis and non-psychosis dimensions.

## 2. Methods

### 2.1. Participants

The present sample consisted of 132 nonclinical young adults (mean age = 27.86, SD = 3.07, range = 26.07, 83% women) belonging to the ongoing Barcelona Longitudinal Investigation of Schizotypy Study (BLISS; Barrantes-Vidal et al., 2013a,b).

At Time 1 (T1), a large pool of 547 unselected college students and 261 technical school students were initially screened with self-report questionnaires. As described in detail elsewhere (Barrantes-Vidal et al., 2013a,b; Torrecilla and Barrantes-Vidal, 2021), a subgroup oversampled for schizotypy scores continued regular follow-ups (from T1 to T5). At T5, 168 (79% of 214 candidate participants) college students and 26 (77% of 31 candidate participants) technical school students were reassessed. Therefore, a total of 194 participants were assessed at T5, when hair samples were collected. From these, 132 participants (112 college students and 20 technical school students) successfully provided 3 cm-hair samples and were included in the present study. Note that sample size may vary for some measures given that three of the participants who provided hair samples did not complete questionnaires at T5 (*N* = 129). All the psychometric measures employed in this study were assessed at T5 along with the cortisol sampling, except early adversity, which was assessed at T1 of the BLISS. All participants provided written informed consent to participate.

### 2.2. Materials and procedure

#### 2.2.1. Measures of adversity and stressful events

To examine early adversity, the Childhood Trauma Questionnaire Short Form (Bernstein et al., 2003) was assessed at T1. CTQ-SF is a self-reported measure including 28 items rating the severity of emotional abuse and neglect, physical abuse and neglect and sexual abuse. A total score of childhood trauma comprising all the subscales is used in the present study (Cronbach’s alpha = 0.85).

Two complementary recent life events measures were used. As a measure of specifically threatening life events, we used The List of Threatening Events (LTE; Brugha and Cragg, 1990). It consists of 20 items (YES/NO) asking about adverse life events that might have occurred during the last year (Cronbach’s alpha = 0.59). As a measure of more general and frequent life events we employed the Life Events Survey (LES; Sarason et al., 1978), which includes 57 life events that might have occurred during the last year comprising a full range of experiences from negative to positive plus three blank spaces for other events (Cronbach’s alpha = 0.78). Forty-seven of them refer to general life events and 10 of them are academic-related. We removed one academic-related item (“Academic probation”) given that there is not an equivalent of it in the Spanish education system. Participants were asked to rate the occurrence (YES/NO) of each life event and the total sum of events endorsed was used. Additionally, the LES also allows to rate the subjective impact of each endorsed life event by using a Likert scale ranging from −3 (extremely negative) to +3 (extremely positive). The sum of ratings from −3 to −1 (negative spectrum) of endorsed life events was used as the amount of negative impact, whereas the sum of ratings from 1 to 3 (positive spectrum) was used as the amount of positive impact. To ease interpretation of results, negative ratings (−3 to −1) were inversely recoded (3 to 1).

#### 2.2.2. Current appraisals of stress

The Perceived Stress Scale (PSS; Cohen et al., 1983) is a self-reported questionnaire of 14 items enquiring about the level of stress perceived by participants during the last month that provides a total score of perceived stress (Cronbach’s alpha = 0.86).

#### 2.2.3. Hair cortisol

To determine HCC, 3 cm of hair strands were cut from the base of the scalp. Considering average hair growth of 1 cm per month (Wennig, 2000), the samples represented the cortisol mean levels from the last 3 months. 40 mg of hair were weighed and washed twice with 4 mL of 2-propanol (SIGMA, Ref: 335639–2.5 L-M) and three extractions were performed overnight with 1.6 mL of methanol (SIGMA, Ref: 34860–2.5 L-M). Methanol was then evaporated in the Speed Vac. The samples were reconstituted with 200 μL of 0.1 M phosphate buffer and then processed in the assay (25 μL in duplicate).

HCC were determined after the above extraction using the Salivary cortisol enzyme immunoassay kit, Expanded Range High Sensitivity (Salimetrics, Ref: 1-3002-5, UK). In brief, cortisol in standards and samples competes with cortisol conjugated to horseradish peroxidase for the cortisol antibody binding sites on a microtiter plate. Bound cortisol-enzyme conjugate is measured by the reaction of the horseradish peroxidase enzyme with the substrate tetramethylbenzidine (blue color), resulting in a yellow color whose optical density is read at 450 nm. Dilution of samples showed good parallelism with the standard curve and recovery of spiking samples was around 100%. In our conditions, the intra-assay coefficient of variation (CV) was 5.1% and the inter-assay CV 6.7%. Nevertheless, all samples were processed within the same assay to avoid inter-assay variability. Participants reported whether they have ever dyed their hair and how many times do they wash their hair weekly.

#### 2.2.4. Phenotype measures

Paranoia was assessed using the Suspiciousness scale (Cronbach’s alpha = 0.70) of the Schizotypal Personality Questionnaire (SPQ: Raine, 1991) and schizotypy was assessed with the short forms of the Wisconsin Schizotypy Scales (WSS-S; Winterstein et al., 2011), from which participants were assigned positive and negative schizotypy factor scores (see Barrantes-Vidal et al., 2013a,b).

Depressive symptoms were assessed with the Beck Depression Inventory-II (BDI-II; Cronbach’s alpha = 0.88) (Beck et al., 1996) and Anxiety was assessed with the Anxiety subscale (Cronbach’s alpha = 0.82) of the Symptom Checklist-90-Revised (SCL-90-R; Derogatis, 1994).

### 2.3. Statistical analyses

ANOVA was used to test differences in hair cortisol concentrations for academic group, sex, age, and hair dye. Hierarchical linear regressions were computed to examine direct and interaction effects of HCC with early and recent exposures on subclinical experiences. HCC and environmental measures were entered at first step and the interaction of HCC with the different environmental measures was entered at the second step. The standardized regression coefficient (β), change in R2, and effect size f2 were reported for each predictor in the regressions. Following Cohen (1992), *f*^2^ values above 0.15 are medium and above 0.35 are large effect sizes. The False Discovery Rate (FDR; Benjamini and Hochberg, 1995) procedure was employed to correct interaction effects for multiple testing across each environmental measure. Finally, simple slope analyses using PROCESS (Hayes, 2013) were computed to decompose significant interactions (*p-*value < 0.05). Statistical analyses were performed using the Statistical Package for the Social Sciences (SPSS), Version 22.0 software (Corp Released, IBM, 2013).

Ethical approval was granted by the Ethics Committee of the Universitat Autònoma de Barcelona. The study was developed in accordance with the Declaration of Helsinki for ethical conduct in research.

## 3. Results

One out of 132 participants was excluded because of abnormally increased HCC (244.6 pg./mg) compared to the rest of the sample. There were not significant differences in mean HCC (*p* = 0.20) among men (*M* = 8.20 pg./mg, SD = 8.31) and women (*M* = 5.81 pg./mg, SD = 3.86), nor between college (*M* = 6.45, SD = 5.17) and technical school (*M* = 4.98, SD = 3.15) students (*p* = 0.22). HCC were not affected by hair dye (*p* = 0.542) or frequency of washing (*p* = 0.146).

**Figure 1:**
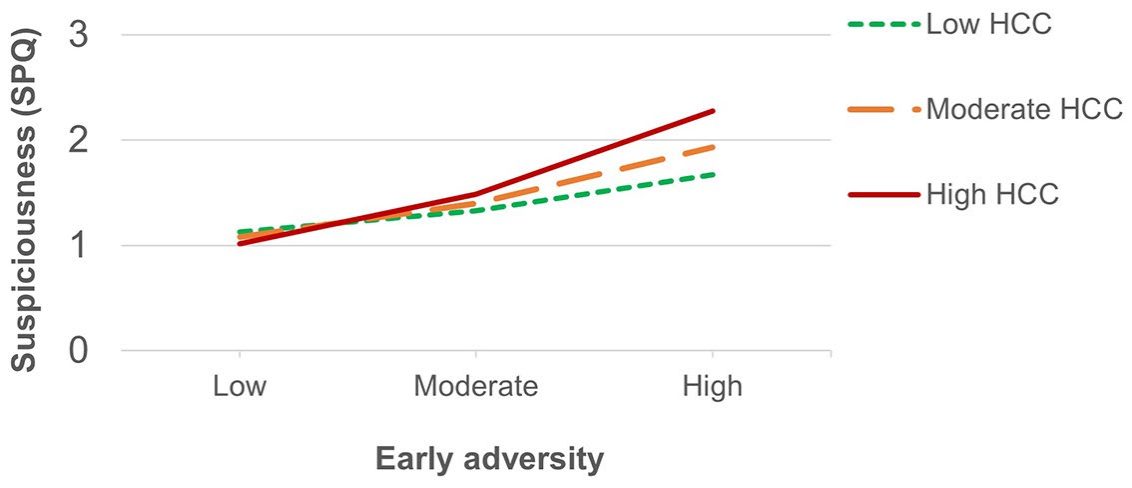
Significant interaction between HCC and early adversity on suspiciousness.

Table 1 shows descriptive statistics and Pearson correlations among study variables. Hierarchical linear regressions results are shown in Tables 2–6. As shown in previous work (Torrecilla and Barrantes-Vidal, 2021), no significant main effects for HCC on any of the stress-related outcomes were detected. Early adversity (Table 2) showed a main effect on all stress-related phenotypes except for negative schizotypy, as well as an interaction with HCC in predicting levels of suspiciousness—a trend-level interaction was found for positive schizotypy. Simple slopes analysis of the significant interaction on suspiciousness (Figure 1) revealed that childhood adversity increased levels of suspiciousness only at high (*β* = 0.43, *p* < 0.01) and moderate (*β* = 0.28, *p* < 0.01) levels of HCC. However, this interaction effect did not survive FDR correction.

**Table 1:** Descriptive statistics and Pearson correlations of the study variables.

	Descriptive statistics			Pearson correlations										
	*N*	*M* (SD)	Range	2	3	4	5	6	7	8	9	10	11	12
1. HCC	131	1.30 (4.94)	1.3–39.7	0.094	0.089	0.102	0.045	0.120	0.089	0.096	0.149	−0.006	−0.046	0.004
2. Early adversity	131	33.91 (8.03)	25–67		0.166	0.203*	0.057	0.229**	195*	0.119	0.348**	0.263**	0.279**	0.284**
3. Threatening life events	128	2.27 (1.98)	0–9			0.371**	0.228**	0.207*	0.262**	−0.070	0.142	−0.012	0.005	0.072
4. General life events	129	10.33 (4.91)	2–24				0.685**	0.574**	0.254**	−0.064	0.172	0.074	0.064	0.114
5. Positive impact events	129	12.89 (9.14)	0–50					−0.056	0.326**	−0.096	0.063	−0.236**	−0.214*	−0.067
6. Negative impact events	129	7.57 (5.9)	0–27						−0.001	−0.060	0.123	0.295**	0.305**	0.217*
7. Positive schizotypy	128	−0.66 (0.45)	−1.17 to 1.32							0.207*	0.473**	0.181*	0.214*	0.262**
8. Negative schizotypy	128	−0.06 (0.92)	−1.03 to 3.26								0.331**	0.243**	0.334**	0.124
9. Suspiciousness	128	1.58 (1.68)	0–8									0.492**	0.511**	0.546**
10. Perceived stress	128	20.19 (7.63)	5–43										0.736**	0.663**
11. Depression	128	5.70 (6.66)	0–35											0.631**
12. Anxiety	128	5.41 (4.75)	0–19											

HCC, Hair Cortisol Concentrations. *p < 0.05; **p < 0.01.

Sample size may vary for some measures given that three of the participants who provided hair samples (*N* = 132) did not fully complete the questionnaire assessment at T5 and one participant was excluded for abnormally increased HCC.

**Table 2:** Main effects of HCC, early adversity, and their interaction on stress-related outcomes.

	Step 1						Step 2		
	Hair cortisol concentrations (HCC)			Early adversity			HCC × early adversity		
	*β*	*ΔR*^2^	*f*^2^	*β*	*ΔR*^2^	*f*^2^	*β*	*ΔR*^2^	*f*^2^
Positive schizotypy	0.073	0.005	0.005	0.189*	0.035	0.036	0.164^+^	0.027	0.029
Negative schizotypy	0.086	0.007	0.008	0.112	0.012	0.013	0.040	0.002	0.001
Suspiciousness	0.120	0.014	0.016	0.337***	0.113	0.130	0.191*	0.036	0.043
Perceived stress	−0.028	0.001	0.001	0.266**	0.070	0.075	0.003	0.000	0.000
Depression	−0.070	0.005	0.005	0.285**	0.081	0.088	−0.099	0.010	0.011
Anxiety	−0.020	0.000	0.001	0.285**	0.081	0.088	0.020	0.000	0.000

^+^*p* < 0.10; *p < 0.05; **p < 0.01; ***p < 0.001.

**Table 3:** Main effects of HCC, recent threatening life events, and their interaction on stress-related outcomes.

	Step 1						Step 2		
	Hair cortisol concentrations (HCC)			Threatening life events			HCC × threatening life events		
	*β*	*ΔR*^2^	*f*^2^	*β*	*ΔR*^2^	*f^2^*	*β*	*ΔR*^2^	*f*^2^
Positive schizotypy	0.067	0.004	0.004	0.256**	0.065	0.070	0.083	0.007	0.007
Negative schizotypy	0.103	0.011	0.010	−0.079	0.006	0.006	0.137	0.018	0.019
Suspiciousness	0.137	0.019	0.019	0.130	0.017	0.017	0.241**^a^	0.058	0.063
Perceived stress	−0.005	0.000	0.000	−0.011	0.000	0.000	0.152^+^	0.023	0.023
Depression	−0.047	0.002	0.001	0.009	0.000	0.000	0.125	0.016	0.016
Anxiety	−0.002	0.000	0.000	0.072	0.005	0.004	0.118	0.014	0.014

^+^*p* < 0.10; **p < 0.01.

a Findings survived FDR correction for multiple comparisons.

**Table 4:** Main effects of HCC, recent general life events, and their interaction on stress-related outcomes.

	Step 1						Step 2		
	Hair cortisol concentrations (HCC)			General life events			HCC × general life events		
	*β*	*ΔR*^2^	*f*^2^	*β*	*ΔR*^2^	*f*^2^	*β*	*ΔR*^2^	*f*^2^
Positive schizotypy	0.064	0.004	0.004	0.248**	0.061	0.065	0.070	0.005	0.005
Negative schizotypy	0.104	0.011	0.011	−0.075	0.006	0.006	−0.064	0.004	0.004
Suspiciousness	0.132	0.017	0.017	159^+^	0.025	0.026	0.209*	0.043	0.047
Perceived stress	−0.019	0.000	0.001	0.075	0.006	0.005	0.180*	0.032	0.033
Depression	−0.054	0.003	0.003	0.075	0.006	0.006	0.129	0.016	0.016
Anxiety	−0.008	0.000	0.000	0.115	0.013	0.013	0.154^+^	0.023	0.023

^+^*p* < 0.10; *p < 0.05; **p < 0.01.

**Table 5:** Main effects of HCC, positive impact of life events, and their interaction on stress-related outcomes.

	Step 1						Step 2		
	Hair cortisol concentrations (HCC)			Positive impact of life events			HCC × positive impact of life events		
	*β*	*ΔR*^2^	*f*^2^	*β*	*ΔR*^2^	*f*^2^	*β*	*ΔR*^2^	*f*^2^
Positive schizotypy	0.075	0.006	0.006	0.323***	0.104	0.117	0.123	0.013	0.014
Negative schizotypy	0.100	0.010	0.010	−0.100	0.010	0.010	−0.092	0.007	0.007
Suspiciousness	0.146	0.021	0.021	0.056	0.003	0.003	0.075	0.005	0.005
Perceived stress	0.005	0.000	0.000	−0.236**	0.056	0.059	266**^a^	0.060	0.067
Depression	−0.036	0.001	0.001	−0.212*	0.045	0.047	0.229*^a^	0.045	0.049
Anxiety	0.007	0.000	0.001	−0.067	0.005	0.005	0.133	0.015	0.015

*p < 0.05; **p < 0.01; ***p < 0.001.

a Findings survived FDR correction for multiple comparisons.

**Table 6:** Main effects of HCC, negative impact of life events, and their interaction on stress-related outcomes.

	Step 1						Step 2		
	Hair cortisol concentrations (HCC)			Negative impact of life events			HCC × negative impact of life events		
	*β*	*ΔR*^2^	*f*^2^	*β*	*ΔR*^2^	*f*^2^	*β*	*ΔR*^2^	*f*^2^
Positive schizotypy	0.091	0.008	0.007	−0.012	0.000	0.000	0.015	0.000	0.000
Negative schizotypy	0.105	0.011	0.010	−0.073	0.005	0.005	−0.071	0.005	0.005
Suspiciousness	0.136	0.018	0.018	0.106	0.011	0.011	0.110	0.011	0.011
Perceived stress	−0.042	0.002	0.002	0.300***	0.088	0.097	0.013	0.000	0.000
Depression	−0.084	0.007	0.007	0.315***	0.098	0.108	−0.001	0.000	0.000
Anxiety	−0.022	0.000	0.001	0.220*	0.048	0.050	0.065	0.004	0.003

*p < 0.05; ***p < 0.001.

**Figure 2:**
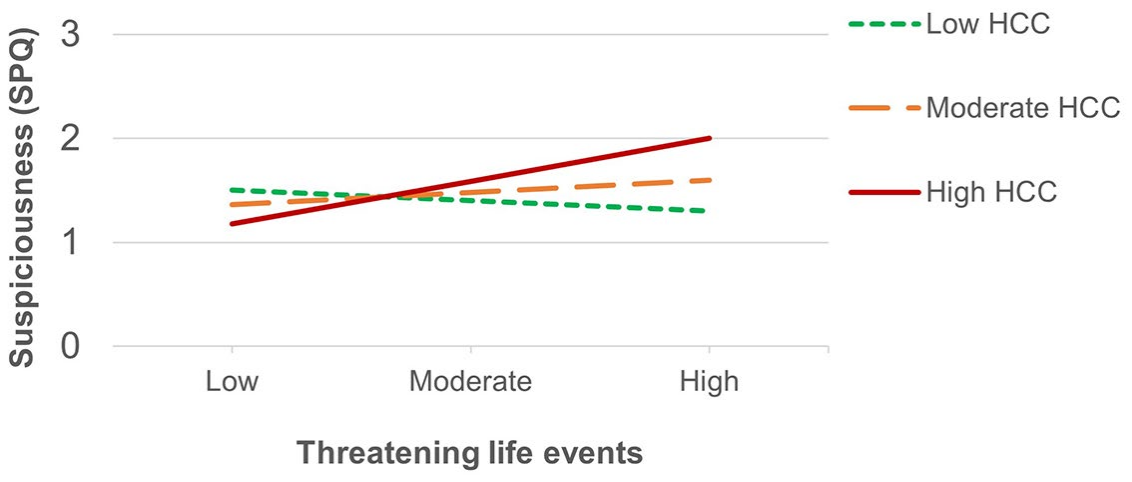
Significant interaction between HCC and recent threatening life events on suspiciousness.

**Figure 3:**
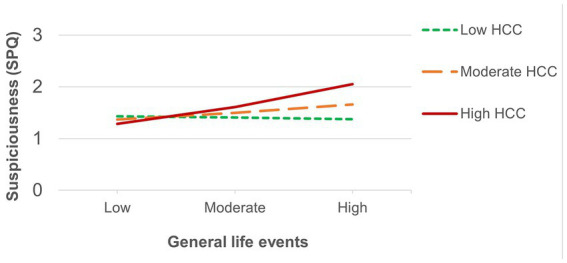
Significant interactions between HCC and recent general life events on suspiciousness.

The number of recent threatening life events was directly associated with positive schizotypy (Table 3) and showed an interaction effect that survived FDR correction with HCC predicting suspiciousness (also for appraisals of perceived stress at a trend level). Simple slopes analysis of the significant interaction on suspiciousness (Figure 2) revealed that threatening life events predicted higher levels of suspiciousness only at high levels of HCC (*β* = 0.24, *p* < 0.05). A similar pattern was found for the measure of general life events (Table 4), showing direct effects on positive schizotypy and statistically significant interactions with HCC for both suspiciousness and perceived stress. However, these interactions did not survive FDR correction. Subsequent simple slopes showed that the general life events increased suspiciousness (Figure 3) only at high levels of HCC (*β* = 0.24, *p* < 0.05), whereas none of the slopes of HCC (low, moderate, high) were significant in the association between HCC and perceived stress.

**Figure 4:**
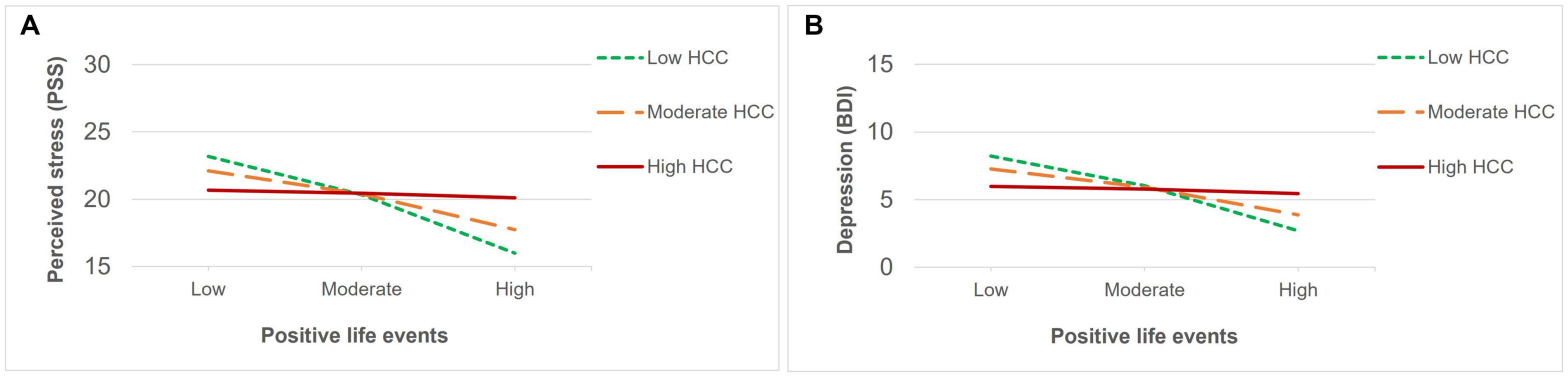
Significant interactions between HCC and recent positive life events on **(A)** perceived stress and **(B)** depression.

Regarding the subjective impact of general life events, positive appraisals (Table 5) were associated with lower levels of perceived stress and depression, but to greater levels of positive schizotypy. In contrast, negative (Table 6) appraisals were associated with greater perceived stress and depression. Interaction effects between HCC and a positive impact of life events (Table 5) were only found for perceived stress and depression, and were significant after multiple testing correction. Both associations (Figures 4A,B) were significant for low (*β* = −0.49, *p* < 0.001 for perceived stress; *β* = −0.43, *p* < 001 for depression) and moderate (*β* = −0.30, *p* < 0.001 for perceived stress; *β* = −0.26, *p* > 0.01 for depression) levels of HCC, indicating that those with lower levels of HCC reported decreased perceived stress and depressive symptoms when they had experienced more positive life events compared to individuals with high HCC who were not affected by the number of positive events. In contrast, no interaction effects between HCC and negative impact of life events were found (Table 6).

## 4. Discussion

This is the first study to examine the moderating role of hair cortisol as a proxy of HPA dysfunction in the association between early and recent stress with a range of stress-related subclinical phenotypes in young adults. HCC consistently moderated the effects of both early and recent life stress on suspiciousness and the effects of recent life events on perceived appraisals of stress. The subjectively rated positive impact of life events was associated with decreases in perceived stress and depression, and these relationships were also moderated by HCC, such that this buffering effect only occurred for participants with low and moderate levels of HCC. Although only interaction effects between HCC and threatening as well as positive life events survived multiple testing correction, these findings provide partial support to the hypothesis that individuals with an hyperresponsive HPA axis present stronger associations between stressors and a variety of subclinical phenotypes robustly associated to stress.

Consistent with evidence suggesting that the positive dimension of psychosis is more strongly associated with childhood trauma (Velikonja et al., 2015; Gibson et al., 2016), early adversity predicted increased positive, but not negative, psychotic-like features, as well as depression, anxiety and increased appraisals of current stress, thus supporting the role of early negative environmental influences on the development of a variety of subclinical psychopathology manifestations (McLaughlin, 2016; Conway et al., 2018). HCC moderated the effects of childhood adversity and recent stressful life events (both general and threatening life events) on suspiciousness. In all cases, experiencing greater levels of early or recent life stress increased levels of suspiciousness specifically in those presenting elevated HCC. These findings are consistent with previous evidence linking childhood trauma as well as recent stress with the HPA axis function, psychiatric illness in general (Murphy et al., 2022) and psychosis in particular (Faravelli et al., 2017; Aas et al., 2019; Herane-Vives et al., 2020). More so, results provide further support for the neural diathesis-stress model (Walker et al., 2008) and extend findings to nonclinical expressions of psychosis liability. The fact that suspiciousness was associated with all forms of stress aligns with cognitive theories suggesting that paranoid thoughts develop as a defensive self-protection mechanism that may arise as a psychological response to threatening or stressful experiences (Freeman and Fowler, 2009). This response was particularly evident in nonclinical individuals whose HPA axis might be disrupted. Of note, HCC also moderated the association between early adversity and positive schizotypy, although only at a trend level. The fact that HCC and childhood trauma only showed interaction effects on psychosis-spectrum features suggests an increased sensitivity of the HPA axis to the effects of early environmental influences for the psychosis spectrum, which supports notions of a general psychopathology severity continuum in which psychotic expressions might index a greater level of severity (McGorry and van Os, 2013; van Os et al., 2020).

Results showed a similar pattern of results for threatening and general recent life events. Both predicted increased levels of positive, but not negative, schizotypy, which is consistent with previous evidence supporting the link between increased stress-sensitivity and positive features of psychosis liability (Kocsis-Bogár et al., 2013; Cristóbal-Narváez et al., 2016; Grattan and Linscott, 2019). HCC moderated the effects of threatening and general life events on suspiciousness and current appraisals of perceived stress, although the latter only reached a trend level for the effects of threatening life events. This slight difference of the effects of threatening events (LTE) compared to general life events (LES) on perceived stress might be related to the greater skewness of the LTE measure in the present sample. As would be expected, most participants may have not encountered, or not as frequently, the severe and threatening experiences (e.g., “Serious illness, injury or assault to self” or “Parent, child or spouse died”) asked in the LTE (*M* = 2.27, SD = 1.98). In contrast, the broader and more generally occurring life events appearing in the LES (e.g., “Major change in sleeping habits,” “Outstanding personal achievement”) are more frequently endorsed by young adults (*M* = 10.33, SD = 4.91) and thus increase the likelihood of detecting possible interaction effects. Of note, this finding also indicates that minor adversities or life-changes are also associated to subclinical features in those with a hyperactivated HPA axis, and suggests the relevance of taking into consideration the developmental phase of study populations when assessing stressful experiences for further research.

Contrary to our expectation, life events subjectively rated as negative did not show any significant interaction effects. In contrast, life events whose impact was rated as positive interacted with HCC to predict levels of perceived stress and depression. Importantly, results showed that experiencing more positive life events significantly decreased appraisals of stress and depressive symptoms, specifically in those with lower levels of HCC, suggesting that positive life events may only exert a protective effect in those who are not biologically sensitized (i.e., showing low HCC). This finding highlights the importance of examining the combined effect of both early and recent experiences of stress as theorized by the stress sensitization hypothesis. Still, though, findings are consistent with emerging literature on the effect of positive environmental influences (Coughlan et al., 2020) as well as positive psychology interventions (Grant et al., 2018b; Grattan and Linscott, 2019) in reducing (the likelihood of) psychopathological outcomes. Interestingly, experiencing positive life events showed a main effect predicting increased positive schizotypy, which resonates with the notion of “happy schizotypes” (Mohr and Claridge, 2015). It has been shown that a proportion of nonclinical individuals with high levels of positive schizotypy (and low in negative and disorganized dimensions) also present hypomanic traits (Kemp et al., 2018) and might reflect “benign schizotypy,” in which psychotic-like experiences are not distressing and might even be rewarding. In fact, as reported in a previous study (Grant and Hennig, 2020), “happy schizotypes” experience psychotic-like experiences in association to momentary happiness.

Lack of significant interactions with the negative impact of life events does not necessarily indicate that experiencing negative stressful situations do not affect the association between the HPA axis function and psychopathology but that probably the effects are not as devastating as we would expect them to be in a clinical or functionally impaired sample, whereas stronger or persistent negative experiences might be needed to capture the effects in a nonclinical sample of functional young adults (Stalder et al., 2017; Torrecilla and Barrantes-Vidal, 2021).

Several strengths characterize the present study. A major advantage compared to the extant literature is the examination of a comprehensive range of psychosocial stressors. We examined both early (i.e., childhood) and recent (i.e., past 12 months) indicators of stress and two complementary measures of recent life events were employed to capture the objective frequency of threatening or uncommon experiences (LTE) as well as more general and commonly faced situations (LES). In addition, we assessed the positive vs. negative subjectively rated impact of general life events in order to better understand individual differences in response to stress (Smith and Pollak, 2020), which has been suggested to result from the individual’s subjective interpretation and appraisal of it rather than the event itself (Lazarus and Folkman, 1984; Thomas et al., 2019). Moreover, we employed a transphenomic approach by assessing traits and subclinical symptoms belonging to the psychotic, affective and anxiety spectrums. This is consistent with the developmental psychopathology notion of multifinality; that is, that the same risk factors can yield differential outcomes according to dynamic transactions with the environment and endogenous factors (Rutter, 2005), and with recent claims about the need to investigate the etiological factors from a multidimensional psychopathology framework to provide further insights on common risk and protective factors across diagnostic spectra (Lynch et al., 2021). Studying a nonclinical sample may have limited the ability to detect the expected effects as nonclinical individuals have probably not been exposed to very high or extreme levels of stress. However, employing a nonclinical sample with significant variance along schizotypy dimensions offers a promising strategy for studying the possible HPA axis dysfunctions as the many confounds associated with clinical status and medication are avoided. Although we provided theory-grounded hypotheses for the analyses conducted, this was primarily an exploratory study given that no previous work has examined this particular moderating effect using the range of outcome measures employed, specifically in the context of psychosis liability (that is, schizotypy). Also, our sample size was limited, and this possibly decreased the ability of detecting interaction effects with sufficient statistical power. Future studies with greater sample sizes that also examine this broad range of relevant stress-related constructs should be higher powered to detect interaction effects that remain after multiple testing correction. The available sample size prevented us from testing a three-way interaction of early and recent stress with cortisol as a moderator to examine how individuals with elevated early-life stress currently facing stressful life events presented with greater subclinical symptoms if biologically sensitized to stress (i.e., with elevated HCC). Future studies with larger sample sizes will also be able to test this and provide further insights on the biological manifestation of the stress sensitization hypothesis and its implications in several psychopathology outcomes. Potential confounders related to HCC such us hair dye, washing frequency, age and sex were examined and did not show any significant associations; however, evidence suggests that other variables such us body mass index should also be considered (Stalder and Kirschbaum, 2012; Stalder et al., 2017). Finally, current dimensional models of early adversity suggest that different dimensions of adversity might have differential effects on psychopathology (McLaughlin and Sheridan, 2016); however, due to limited statistical power, the present study only examined a general total score of trauma. Further and better powered studies will be able to examine differences across subtypes and co-occurrence of adverse experiences.

To conclude, the present findings support the moderating role of retrospective long-term levels of cortisol (i.e., HCC) on the association with both early and recent stressful experiences for a wide range of subclinical measures. Also, the buffering effects of recent positive experiences contribute to reduce pessimism around subclinical psychological manifestations and supports the importance of further focusing on positive resilience-building early interventions.

## Data availability statement

The raw data supporting the conclusions of this article will be made available by the authors, without undue reservation.

## Ethics statement

The studies involving human participants were reviewed and approved by Ethics Committee of the Universitat Autònoma de Barcelona [Comissió d’Ètica en l’Experimentació Animal i Humana (CEEAH)]. The patients/participants provided their written informed consent to participate in this study.

## Author contributions

NB-V conceived the study, acquired funding, administered and supervised the project and data acquisition, contributed and revised the manuscript. PT analyzed the data and wrote the manuscript. All authors contributed to the article and approved the submitted version.

## Funding

This work was supported by the Spanish Ministry of Science, Innovation and Universities (Grants numbers PSI2017-87512-C2-1-R; PID2020-11921RB-I00) and the Comissionat per a Universitats i Recerca of Generalitat de Catalunya (Grant number 2021SGR01010). PT is supported by the Spanish Ministry of Science, Innovation and Universities (Grant number PRE2018-085299). NB-V is supported by the ICREA Academia Award of the Generalitat de Catalunya.

## Conflict of interest

The authors declare that the research was conducted in the absence of any commercial or financial relationships that could be construed as a potential conflict of interest.

## Publisher’s note

All claims expressed in this article are solely those of the authors and do not necessarily represent those of their affiliated organizations, or those of the publisher, the editors and the reviewers. Any product that may be evaluated in this article, or claim that may be made by its manufacturer, is not guaranteed or endorsed by the publisher.

## References

[ref1] Aas M. Pizzagalli D. A. Laskemoen J. F. Reponen E. J. Ueland T. Melle I.. (2019). Elevated hair cortisol is associated with childhood maltreatment and cognitive impairment in schizophrenia and in bipolar disorders. Schiz. Res. 213, 65–71. doi: 10.1016/j.schres.2019.01.011, PMID: 30660575

[ref2] Anderson L. R. Monden C. W. S. Bukodi E. (2022). Stressful life events, differential vulnerability, and depressive symptoms: critique and new evidence. J. Health Soc. Behav. 63, 283–300. doi: 10.1177/00221465211055993, PMID: 34809472 PMC9136473

[ref3] Andrade E. H. Rizzo L. B. Noto C. Ota V. K. Gadelha A. Daruy-Filho L.. (2016). Hair cortisol in drug-naïve first-episode individuals with psychosis. Braz. J. Psychatr. 38, 11–16. doi: 10.1590/1516-4446-2014-1634, PMID: 26814837 PMC7115472

[ref4] Bandoli G. Campbell-Sills L. Kessler R. C. Heeringa S. G. Nock M. K. Rosellini A. J.. (2017). Childhood adversity, adult stress, and the risk of major depression or generalized anxiety disorder in US soldiers: a test of the stress sensitization hypothesis. Psychol. Med. 47, 2379–2392. doi: 10.1017/S0033291717001064, PMID: 28443533 PMC5595661

[ref5] Barrantes-Vidal N. Chun C. A. Myin-Germeys I. Kwapil T. R. (2013a). Psychometric schizotypy predicts psychotic-like, paranoid, and negative symptoms in daily life. J. Ab. Psychol. 122, 1077–1087. doi: 10.1037/a0034793, PMID: 24364610

[ref6] Barrantes-Vidal N. Gross G. M. Sheinbaum T. Mitjavila M. Ballespí S. Kwapil T. R. (2013b). Positive and negative schizotypy are associated with prodromal and schizophrenia-spectrum symptoms. Schiz. Res. 145, 50–55. doi: 10.1016/j.schres.2013.01.007, PMID: 23402694

[ref7] Beck A. T. Steer R. A. Ball R. Ranieri W. (1996). Comparison of Beck depression inventories -IA and -II in psychiatric outpatients. J. Pers. Assessm. 67, 588–597. doi: 10.1207/s15327752jpa6703_13, PMID: 8991972

[ref8] Belda X. Fuentes S. Daviu N. Nadal R. Armario A. (2015). Stress-induced sensitization: the hypothalamic-pituitary-adrenal axis and beyond. Stress 18, 269–279. doi: 10.3109/10253890.2015.1067678, PMID: 26300109

[ref9] Benjamini Y. Hochberg Y. (1995). Controlling the false discovery rate: a practical and powerful approach to multiple testing. J. R. Stat. Soc. B Stat. Methodol. 57, 289–300. doi: 10.1111/j.2517-6161.1995.tb02031.x

[ref10] Bernstein D. P. Stein J. A. Newcomb M. D. Walker E. Pogge D. Ahluvalia T.. (2003). Development and validation of a brief screening version of the childhood trauma questionnaire. Child Abuse Negl. 27, 169–190. doi: 10.1016/S0145-2134(02)00541-0, PMID: 12615092

[ref12] Brugha T. S. Cragg D. (1990). The list of threatening experiences: the reliability and validity of a brief life events questionnaire. Acta Psychiatr. Scand. 82, 77–81. doi: 10.1111/j.1600-0447.1990.tb01360.x, PMID: 2399824

[ref13] Cohen J. (1992). A power primer. Psychol. Bull. 112, 155–159. doi: 10.1037/0033-2909.112.1.155 19565683

[ref14] Cohen S. Kamarck T. Mermelstein R. (1983). A global measure of perceived stress. J. Health Soc. Behav. 24, 385–396. doi: 10.2307/2136404 6668417

[ref15] Collip D. Myin-Germeys I. Van Os J. (2008). Does the concept of "sensitization" provide a plausible mechanism for the putative link between the environment and schizophrenia? Schiz. Bull. 34, 220–225. doi: 10.1093/schbul/sbm163, PMID: 18203757 PMC2632409

[ref16] Conway C. C. Raposa E. B. Hammen C. (2018). Brennan PATransdiagnostic pathways from early social stress to psychopathology: a 20-year prospective study. J. Child Psychol. Psych. 59, 855–862. doi: 10.1111/jcpp.12862, PMID: 29315560 PMC12490286

[ref17] Corp Released, IBM. IBM SPSS statistics for windows, Version 22.0. IBM Corp, Armonk, NY. (2013)

[ref18] Coughlan H. Healy C. Ní Sheaghdha Á. Murray G. Humphries N. Clarke M.. (2020). Early risk and protective factors and young adult outcomes in a longitudinal sample of young people with a history of psychotic-like experiences. Early Interv. Psych. 14, 307–320. doi: 10.1111/eip.12855, PMID: 31310453

[ref19] Cristóbal-Narváez P. Sheinbaum T. Ballespí S. Mitjavila M. Myin-Germeys I. Kwapil T. R.. (2016). Impact of adverse childhood experiences on psychotic-like symptoms and stress reactivity in daily life in nonclinical Young adults. PLoS One 11:e0153557. doi: 10.1371/journal.pone.0153557, PMID: 27082442 PMC4833319

[ref20] Cullen A. E. Rai S. Vaghani M. S. Mondelli V. McGuire P. (2020). Cortisol responses to naturally occurring psychosocial stressors across the psychosis Spectrum: a systematic review and Meta-analysis. Front. Psych. 11:513. doi: 10.3389/fpsyt.2020.00513, PMID: 32595532 PMC7300294

[ref21] Derogatis L. SCL-90-R. symptom Checklist-90-R. administration, scoring and procedures manual. Minneapolis: National Computer System (1994).

[ref22] Duncko R. Fischer S. Hatch S. L. Frissa S. Goodwin L. Papadopoulos A.. (2019). Recurrence of depression in relation to history of childhood trauma and hair cortisol concentration in a community-based sample. Neuropsychobiology 78, 48–57. doi: 10.1159/000498920, PMID: 30897568

[ref23] Ettinger U. Meyhöfer I. Steffens M. Wagner M. Koutsouleris N. (2014). Genetics, cognition, and neurobiology of schizotypal personality: a review of the overlap with schizophrenia. Front. Psych. 5:18. doi: 10.3389/fpsyt.2014.00018, PMID: 24600411 PMC3931123

[ref24] Faravelli C. Mansueto G. Palmieri S. Lo Sauro C. Rotella F. Pietrini F.. (2017). Childhood adversity, cortisol levels, and psychosis: a retrospective investigation. J. Nerv. Ment. Dis. 205, 574–579. doi: 10.1097/NMD.0000000000000699 28598957

[ref25] Freeman D. Fowler D. (2009). Routes to psychotic symptoms: trauma, anxiety and psychosis-like experiences. Psychiatry Res. 169, 107–112. doi: 10.1016/j.psychres.2008.07.009, PMID: 19700201 PMC2748122

[ref26] Fuchs A. Jaite C. Neukel C. Dittrich K. Bertsch K. Kluczniok D.. (2018). Link between children's hair cortisol and psychopathology or quality of life moderated by childhood adversity risk. Psychoneuroendocrinology 90, 52–60. doi: 10.1016/j.psyneuen.2018.02.003, PMID: 29433073

[ref27] Gibson L. E. Alloy L. B. Ellman L. M. (2016). Trauma and the psychosis spectrum: a review of symptom specificity and explanatory mechanisms. Clin. Psychol. Rev. 49, 92–105. doi: 10.1016/j.cpr.2016.08.003, PMID: 27632064 PMC5157832

[ref28] Grant P. Green M. J. Mason O. J. (2018a). Models of Schizotypy: the importance of conceptual clarity. Schiz. Bull. 44, S556–S563. doi: 10.1093/schbul/sby012, PMID: 29474661 PMC6188508

[ref29] Grant P. Hennig J. (2020). Schizotypy, social stress and the emergence of psychotic-like states - a case for benign schizotypy? Schiz. Res. 216, 435–442. doi: 10.1016/j.schres.2019.10.052, PMID: 31796309

[ref30] Grant P. Munk A. Hennig J. (2018b). A positive-psychological intervention reduces acute psychosis-proneness. Schiz. Res. 199, 414–419. doi: 10.1016/j.schres.2018.04.007, PMID: 29661523

[ref31] Grattan R. E. Linscott R. J. (2019). Components of schizophrenia liability affect the growth of psychological stress sensitivity following major life events. Schiz. Res. 212, 134–139. doi: 10.1016/j.schres.2019.07.056 31387827

[ref32] Hammen C. L. (2015). Stress and depression: old questions, new approaches. Curr. Opin. Psychol. 4, 80–85. doi: 10.1016/j.copsyc.2014.12.024

[ref33] Hayes AF. Introduction to mediation, moderation, and conditional process analysis: a regression-based approach. New York: Guilford Press, (2013). 56.

[ref34] Herane-Vives A. Young A. H. Wise T. Aguirre J. de Angel V. Arnone D.. (2020). Comparison of short-term (saliva) and long-term (hair) cortisol levels in out-patients with melancholic and non-melancholic major depression. BJPsych Open 6, e41–e48. doi: 10.1192/bjo.2020.8, PMID: 32321622 PMC7189571

[ref35] Kemp K. C. Gross G. M. Barrantes-Vidal N. Kwapil T. R. (2018). Association of positive, negative, and disorganized schizotypy dimensions with affective symptoms and experiences. Psychiatry Res. 270, 1143–1149. doi: 10.1016/j.psychres.2018.10.031, PMID: 30366639

[ref36] Kocsis-Bogár K. Miklósi M. Forintos D. P. (2013). Impact of adverse life events on individuals with low and high schizotypy in a nonpatient sample. J. Nerv. Ment. Dis. 201, 208–215. doi: 10.1097/NMD.0b013e3182845cea 23417012

[ref37] Koumantarou Malisiova E. Mourikis I. Darviri C. Nicolaides N. C. Zervas I. M. Papageorgiou C.. (2021). Hair cortisol concentrations in mental disorders: a systematic review. Physiol. Behav. 229:113244. doi: 10.1016/j.physbeh.2020.113244, PMID: 33181165

[ref38] Kwapil T. R. Barrantes-Vidal N. (2015). Schizotypy: looking back and moving forward. Schiz. Bull. 41, S366–S373. doi: 10.1093/schbul/sbu186, PMID: 25548387 PMC4373633

[ref39] Lataster J. Myin-Germeys I. Lieb R. Wittchen H. U. van Os J. (2012). Adversity and psychosis: a 10-year prospective study investigating synergism between early and recent adversity in psychosis. Acta Psych. Scand. 125, 388–399. doi: 10.1111/j.1600-0447.2011.01805.x, PMID: 22128839

[ref40] Lazarus R. S. Folkman S. (1984). Stress, appraisal, and coping, New York: Springer Publishing Company.

[ref41] Lynch S. J. Sunderland M. Newton N. C. Chapman C. (2021). A systematic review of transdiagnostic risk and protective factors for general and specific psychopathology in young people. Clin. Psychol. Rev. 87:102036. doi: 10.1016/j.cpr.2021.102036, PMID: 33992846

[ref42] McGorry P. D. Hartmann J. A. Spooner R. Nelson B. (2018). Beyond the "at risk mental state" concept: transitioning to transdiagnostic psychiatry. World Psych. 17, 133–142. doi: 10.1002/wps.20514, PMID: 29856558 PMC5980504

[ref43] McGorry P. van Os J. (2013). Redeeming diagnosis in psychiatry: timing versus specificity. Lancet 381, 343–345. doi: 10.1016/S0140-6736(12)61268-9, PMID: 23351805

[ref44] McLaughlin K. A. (2016). Future directions in childhood adversity and youth psychopathology. J. Clin. Child Psychol. 45, 361–382. doi: 10.1080/15374416.2015.1110823, PMID: 26849071 PMC4837019

[ref45] McLaughlin K. A. Conron K. J. Koenen K. C. Gilman S. E. (2010). Childhood adversity, adult stressful life events, and risk of past-year psychiatric disorder: a test of the stress sensitization hypothesis in a population-based sample of adults. Psychol. Med. 40, 1647–1658. doi: 10.1017/S0033291709992121, PMID: 20018126 PMC2891275

[ref46] McLaughlin K. A. Sheridan M. A. (2016). Beyond cumulative risk: a dimensional approach to childhood adversity. Curr. Dir. Psychol. Sci. 25, 239–245. doi: 10.1177/0963721416655883, PMID: 27773969 PMC5070918

[ref47] Mohr C. Claridge G. (2015). Schizotypy--do not worry, it is not all worrisome. Schizophr. Bull. Open 41 Suppl 2, S436–S443. doi: 10.1093/schbul/sbu185, PMID: 25810058 PMC4373632

[ref48] Mondelli V. Dazzan P. (2019). Childhood trauma and psychosis: moving the field forward. Schiz. Res. 205, 1–3. doi: 10.1016/j.schres.2019.02.001, PMID: 30765250

[ref49] Murphy F. Nasa A. Cullinane D. Raajakesary K. Gazzaz A. Sooknarine V.. (2022). Childhood trauma, the HPA Axis and psychiatric illnesses: a targeted literature synthesis. Front. Psych. 13:748372. doi: 10.3389/fpsyt.2022.748372, PMID: 35599780 PMC9120425

[ref50] Myin-Germeys I. van Os J. (2007). Stress-reactivity in psychosis: evidence for an affective pathway to psychosis. Clin. Psychol. Rev. 27, 409–424. doi: 10.1016/j.cpr.2006.09.005, PMID: 17222489

[ref51] Naughton M. Dinan T. G. Scott L. V. (2014, 2014). “Corticotropin-releasing hormone and the hypothalamic-pituitary-adrenal axis in psychiatric disease” in Handbook of clinical neurology. eds. Fliers E. Korbonits M. Romijn J. A. (Toronto: Elsevier), 69–91. 10.1016/B978-0-444-59602-4.00005-8 25248580

[ref52] Pruessner M. Cullen A. E. Aas M. Walker E. F. (2017). The neural diathesis-stress model of schizophrenia revisited: an update on recent findings considering illness stage and neurobiological and methodological complexities. Neurosci. Biobehav. Rev. 73, 191–218. doi: 10.1016/j.neubiorev.2016.12.013, PMID: 27993603

[ref53] Raine A. (1991). The spq: a scale for the assessment of schizotypal personality based on DSM-III-r criteria. Schiz. Bull. 17, 555–564. doi: 10.1093/schbul/17.4.555, PMID: 1805349

[ref54] Rauschenberg C. van Os J. Cremers D. Goedhart M. Schieveld J. N. M. Reininghaus U. (2017). Stress sensitivity as a putative mechanism linking childhood trauma and psychopathology in youth’s daily life. Acta Psychiatr. Scand. 136, 373–388. doi: 10.1111/acps.12775, PMID: 28758672

[ref55] Read J. Perry B. D. Moskowitz A. Connolly J. (2001). The contribution of early traumatic events to schizophrenia in some patients: a traumagenic neurodevelopmental model. Psychiatry 64, 319–345. doi: 10.1521/psyc.64.4.319.18602, PMID: 11822210

[ref56] Russell E. Koren G. Rieder M. Van Uum S. (2012). Hair cortisol as a biological marker of chronic stress: current status, future directions and unanswered questions. Psychoneuroendocrinology 37, 589–601. doi: 10.1016/j.psyneuen.2011.09.009, PMID: 21974976

[ref57] Rutter M. (2005). Multiple meanings of a developmental perspective on psychopathology. Eur. J. Dev. Psychol. 2, 221–252. doi: 10.1080/17405620500237706

[ref58] Sarason I. G. Johnson J. H. Siegel J. M. (1978). Assessing the impact of life changes: development of the life experiences survey. J. Consult. Clin. Psychol. 46, 932–946. doi: 10.1037//0022-006x.46.5.932 701572

[ref59] Schalinski I. Teicher M. H. Rockstroh B. (2019). Early neglect is a key determinant of adult hair cortisol concentration and is associated with increased vulnerability to trauma in a transdiagnostic sample. Psychoneuroendocrinology 108, 35–42. doi: 10.1016/j.psyneuen.2019.06.007, PMID: 31226659

[ref60] Shapero B. G. Curley E. E. Black C. L. Alloy L. B. (2019). The interactive association of proximal life stress and cumulative HPA axis functioning with depressive symptoms. Depress. Anxiety 36, 1089–1101. doi: 10.1002/da.22957 31614065

[ref61] Sideli L. Murray R. M. Schimmenti A. Corso M. La Barbera D. Trotta A.. (2020). Childhood adversity and psychosis: a systematic review of bio-psycho-social mediators and moderators. Psychol. Med. 50, 1761–1782. doi: 10.1017/S0033291720002172, PMID: 32624020

[ref62] Smith K. E. Pollak S. D. (2020). Early life stress and development: potential mechanisms for adverse outcomes. J. Neurodev. Dis. 12:34. doi: 10.1186/s11689-020-09337-y, PMID: 33327939 PMC7745388

[ref63] Söder E. Clamor A. Lincoln T. M. (2019). Hair cortisol concentrations as an indicator of potential HPA axis hyperactivation in risk for psychosis. Schiz. Res. 212, 54–61. doi: 10.1016/j.schres.2019.08.012, PMID: 31455519

[ref64] Stalder T. Kirschbaum C. (2012). Analysis of cortisol in hair--state of the art and future directions. Brain Behav. Immun. 26, 1019–1029. doi: 10.1016/j.bbi.2012.02.002 22366690

[ref65] Stalder T. Steudte-Schmiedgen S. Alexander N. Klucken T. Vater A. Wichmann S.. (2017). Stress-related and basic determinants of hair cortisol in humans: a meta-analysis. Psychoneuroendocrinology 77, 261–274. doi: 10.1016/j.psyneuen.2016.12.017, PMID: 28135674

[ref66] Streit F. Memic A. Hasandedić L. Rietschel L. Frank J. Lang M.. (2016). Perceived stress and hair cortisol: differences in bipolar disorder and schizophrenia. Psychoneuroendocrinology 69, 26–34. doi: 10.1016/j.psyneuen.2016.03.010 27017430

[ref67] Thomas A. J. Mitchell E. S. Woods N. F. (2019). Undesirable stressful life events, impact, and correlates during midlife: observations from the Seattle midlife women's health study. Womens Midlife Health 5:1. doi: 10.1186/s40695-018-0045-y, PMID: 30766725 PMC6318955

[ref68] Torrecilla P. Barrantes-Vidal N. (2021). Examining the relationship between hair cortisol with stress-related and Transdiagnostic subclinical measures. Front. Psych. 12:746155. doi: 10.3389/fpsyt.2021.746155, PMID: 34858226 PMC8631911

[ref69] van Os J. (2015). The transdiagnostic dimension of psychosis: implications for psychiatric nosology and research. Shanghai Arch. 27, 82–86. doi: 10.11919/j.issn.1002-0829.215041, PMID: 26120256 PMC4466847

[ref70] van Os J. Pries L. K. Ten Have M. de Graaf R. van Dorsselaer S. Delespaul P.. (2020). Evidence, and replication thereof, that molecular-genetic and environmental risks for psychosis impact through an affective pathway. Psychol. Med. 52, 1910–1922. doi: 10.1017/S0033291720003748, PMID: 33070791

[ref71] Van Winkel R. Stefanis N. C. Myin-Germeys I. (2008). Psychosocial stress and psychosis. A review of the neurobiological mechanisms and the evidence for gene-stress interaction. Schiz. Bull. 34, 1095–1105. doi: 10.1093/schbul/sbn101, PMID: 18718885 PMC2632486

[ref72] Velikonja T. Fisher H. L. Mason O. Johnson S. (2015). Childhood trauma and schizotypy: a systematic literature review. Psychol. Med. 45, 947–963. doi: 10.1017/S0033291714002086, PMID: 25273151

[ref73] Walker E. F. Diforio D. (1997). Schizophrenia: a neural diathesis-stress model. Psychol. Rev. 104, 667–685. doi: 10.1037/0033-295X.104.4.667, PMID: 9337628

[ref74] Walker E. Mittal V. Tessner K. (2008). Stress and the hypothalamic pituitary adrenal Axis in the developmental course of schizophrenia. Annu. Rev. Clin. Psychol. 4, 189–216. doi: 10.1146/annurev.clinpsy.4.022007.141248, PMID: 18370616

[ref75] Wennig R. (2000). Potential problems with the interpretation of hair analysis results. Forensic Sci. Int. 107, 5–12. doi: 10.1016/S0379-0738(99)00146-2, PMID: 10689559

[ref76] Winterstein B. P. Silvia P. J. Kwapil T. R. Kaufman J. C. Reiter-Palmon R. Wigert B. (2011). Brief assessment of schizotypy: developing short forms of the Wisconsin Schizotypy scales. Pers. Individ. Differ. 51, 920–924. doi: 10.1016/j.paid.2011.07.027

[ref77] Xu Y. Liu Y. Chen Z. Zhang J. Deng H. Gu J. (2019). Interaction effects of life events and hair cortisol on perceived stress, anxiety, and depressive symptoms among Chinese adolescents: testing the differential susceptibility and diathesis-stress models. Front. Psychol. 10, 1–10. doi: 10.3389/fpsyg.2019.00297, PMID: 30890975 PMC6411789

[ref78] Yuii K. Suzuki M. Kurachi M. (2007). Stress sensitization in schizophrenia. Ann. N. Y. Acad. Sci. 1113, 276–290. doi: 10.1196/annals.1391.013 17584979

